# Chronic disease burden associated with overweight and obesity in Ireland: the effects of a small BMI reduction at population level

**DOI:** 10.1186/1471-2458-14-143

**Published:** 2014-02-10

**Authors:** Karen Kearns, Anne Dee, Anthony P Fitzgerald, Edel Doherty, Ivan J Perry

**Affiliations:** 1 Department of Epidemiology and Public Health, University College Cork, Cork, Ireland; 2 HSE Department of Public Health, Mount Kennett House, Henry Street, Limerick, Ireland; 3 J.E. Cairnes School of Business and Economics, National University of Ireland Galway, Galway, Ireland; 4 Department of Statistics, University College Cork, Cork, Ireland; 5 Healthcare Pricing Office, ESRI Building, Whitaker Square, Sir John Rogerson’s Quay, Dublin 2, Ireland

**Keywords:** Overweight, Obesity, BMI, Burden, Chronic disease, Prevalence, Population attributable fraction

## Abstract

**Background:**

Overweight and obesity prevalence has risen dramatically in recent decades. While it is known that overweight and obesity is associated with a wide range of chronic diseases, the cumulative burden of chronic disease in the population associated with overweight and obesity is not well quantified. The aims of this paper were to examine the associations between BMI and chronic disease prevalence; to calculate Population Attributable Fractions (PAFs) associated with overweight and obesity; and to estimate the impact of a one unit reduction in BMI on the population prevalence of chronic disease.

**Methods:**

A cross-sectional analysis of 10,364 adults aged ≥18 years from the Republic of Ireland National Survey of Lifestyle, Attitudes and Nutrition (SLÁN 2007) was performed. Using binary regression, we examined the relationship between BMI and the selected chronic diseases. In further analyses, we calculated PAFs of selected chronic diseases attributable to overweight and obesity and we assessed the impact of a one unit reduction in BMI on the overall burden of chronic disease.

**Results:**

Overweight and obesity prevalence was higher in men (43.0% and 16.1%) compared to women (29.2% and 13.4%), respectively. The most prevalent chronic conditions were lower back pain, hypertension, and raised cholesterol. Prevalence of chronic disease generally increased with increasing BMI. Compared to normal weight persons, the strongest associations were found in obese women for diabetes (RR 3.9, 95% CI 2.5-6.3), followed by hypertension (RR 2.9, 95% CI 2.3-3.6); and in obese men for hypertension (RR 2.1, 95% CI 1.6-2.7), followed by osteoarthritis (RR 2.0, 95% CI 1.2-3.2). Calculated PAFs indicated that a large proportion of chronic disease is attributable to increased BMI, most noticeably for diabetes in women (42%) and for hypertension in men (30%). Overall, a one unit decrease in BMI results in 26 and 28 fewer cases of chronic disease per 1,000 men and women, respectively.

**Conclusions:**

Overweight and obesity are major contributors to the burden of chronic disease in the population. The achievement of a relatively modest reduction in average BMI in the population has the potential to make a significant impact on the burden of chronic disease.

## Background

Globally, the prevalence of overweight and obesity has increased rapidly in recent decades. Global estimates from 2008 show that 1.5 billion adults, 20 years and older, were overweight, and of these over 200 million men and nearly 300 million women were obese [1]. Projected trends suggest that there will be 65 million more obese adults in the United States in 2030 than in 2010, and 11 million more obese adults in the United Kingdom (UK) [2]. Further projections estimate that by 2035, obesity rates in UK adults are estimated to rise to 47% for men and 36% for women, and by 2050, this could rise to 60% for men and 50% for women [3].

Due to these increases, overweight and obesity are now major contributors to the global burden of disease [1,4]. Overweight and obesity are the fifth leading risk for global deaths, resulting in at least 2.8 million adult deaths each year [1]. Estimates from 2004 indicate that 2.3% (35.8 million) of total global disability-adjusted life years (DALYs) were attributed to overweight and obesity [5]. As an increase in the prevalence of overweight and obesity is expected over the next two decades [6], the burden of disease associated with overweight and obesity is likely to increase.

Substantial literature indicates that overweight and obese individuals have an increased risk of developing a number of chronic diseases, which can lead to further morbidity and mortality [7-9], with morbidity having a more pronounced impact [10]. Such chronic diseases include type 2 diabetes, cardiovascular disease (CVD) and cardiovascular risk factors, respiratory diseases such as asthma, musculoskeletal disorders such as osteoarthritis and low back pain, several cancers, and depression [1,11,12].

The high prevalence of overweight and obesity in Ireland, 36% and 14% respectively [13], based on self-reported data, is likely to be contributing to an increase in the overall burden of chronic disease. At present, the cumulative burden of prevalent chronic disease associated with overweight and obesity is not well quantified. This study aimed to investigate the burden of chronic disease associated with overweight and obesity, as defined by Body Mass Index (BMI) category, in the adult population. The specific objectives of this study were: (i) to describe the prevalence of overweight, obesity, and chronic disease (ii) to examine the association between BMI and chronic disease prevalence; (iii) to calculate Population Attributable Fractions (PAFs) associated with overweight and obesity; and (iv) to estimate the impact of a one unit reduction in BMI on the population prevalence of chronic disease.

## Methods

### Ethics statement

SLÁN 2007 was approved by the Research Ethics Committee of the Royal College of Surgeons of Ireland. This study analysed de-identified, secondary data and was therefore exempt from Clinical Research Ethics Committee review.

### Study design and covariates

A cross-sectional analysis was conducted using data from the 2007 Survey of Lifestyle, Attitudes and Nutrition (SLÁN 2007), the most recent of a series of nationally representative health surveys in the Republic of Ireland. The main component of the SLÁN 2007 survey consisted of face-to-face interviews conducted with 10,364 randomly selected participants (response rate 62%). The survey also included measurements of height, weight, and waist circumference for 967 respondents aged 18–44 years and a detailed physical examination of 1,207 respondents aged 45 years and over. Given the small number of respondents for which measured BMI was collected, self-reported BMI was considered a better measure to use given the much larger sample size. Self-reported height and weight were used to calculate BMI for 9,725 respondents. The population for SLÁN 2007 was defined as adults aged 18 years and over, living in private households in the Republic of Ireland. The survey consisted of a probabilistic sample in three stages - geographic area, household and ‘next birthday’ participant selection within households. The sample was drawn from the Geodirectory, a listing of all residential addresses in Ireland compiled by the postal service. Further details on study design and sampling can be found elsewhere [13]. SLÁN 2007 measured socio-demographic variables including gender, age, highest level of education attained, marital and employment status, residential location, annual household income, and social class. The social class scheme assigns individuals and households to social class groups according to occupation and is based on the European Socio-economic Classification (ESeC) [14]. The survey also measured lifestyle behaviours including diet and nutrition, physical activity, alcohol and smoking status.

Chronic disease was based on self-reported occurrence, based on the question “Have you had any of the following in the last 12 months?”. Chronic diseases included in this analysis were lower back pain, osteoarthritis, diabetes mellitus, CVD (includes stroke, heart attack, angina), asthma, bronchitis (includes chronic bronchitis, chronic obstructive pulmonary disease, emphysema), anxiety, and depression. Cardiovascular risk factors included in the analysis were hypertension and raised cholesterol. These were based on the question “In the last 12 months, have you been screened or tested for any of the following?”. As the prevalence of stroke, heart attack, and angina were low, 0.8%, 1.0%, and 2.2% respectively, they were combined as one variable, CVD, for the analyses. Self- reported height and weight were used to calculate BMI as a measure of overweight and obesity. BMI was categorised into four groups: underweight 15.0-18.49 kg/m^2^, normal weight 18.5-24.99 kg/m^2^, overweight 25.0-29.99 kg/m^2^, and obese ≥30 kg/m^2^. Due to the relatively small numbers involved, the obese category was not further sub-divided (n = 1,241). The underweight category was excluded from the analyses due to the small numbers in this category (n = 182). The following socio-economic and lifestyle variables were included in the association analysis: age (18–24; 25–34; 35–44; 45–54; 55–64; and 65+ years), highest level of education attained (primary; secondary; third level), social class (classes 1–5, ranging from highest (class 1 i.e. ESeC 1–2) to lowest (class 4 i.e. ESeC 7–9), with class 5 as never worked/unclassified/unknown (i.e. ESeC 10)), employment status (employed; unemployed), type of smoker (current smoker; former/never smoker), and frequency of drinking alcohol (never; moderate i.e. ‘monthly or less’ or ‘3 to 4 times a month’; frequent i.e. ‘2-3 times a week’ or ‘4 or more times a week’).

### Statistical analyses

All analyses were carried out using the statistical software program Stata, version 12.0 [15]. Sampling weights were applied to take into account any differences in the characteristics of the survey sample compared to the population of interest. BMI was considered both as a categorical and a continuous variable. Descriptive statistics by BMI category, including age, socio-economic characteristics and lifestyle behaviours are presented in Table 1. Statistical significance was defined as P < 0.05.

**Table 1:** Socio-demographic and other selected characteristics, n (%), by BMI category and gender

	**Males (n = 4,260)**			**Females (n = 4,171)**		
	**Normal weight**	**Overweight**	**Obese**	**Normal weight**	**Overweight**	**Obese**
All ages	1,745 (41.0)	1,831 (43.0)	684 (16.1)	2,397 (57.5)	1,217 (29.2)	557 (13.4)
No chronic disease	1,272 (73.7)	1,248 (69.4)	446 (66.3)	1,650 (69.7)	777 (64.7)	305 (55.6)
1 chronic disease	335 (19.4)	403 (22.4)	151 (22.5)	503 (21.2)	268 (22.3)	148 (27.0)
2+ chronic diseases	119 (6.9)	147 (8.2)	76 (11.2)	215 (9.1)	156 (13.0)	96 (17.4)
Age (years)						
18–24	438 (25.1)	136 (7.4)	25 (3.7)	443 (18.5)	78 (6.4)	45 (8.1)
25–34	464 (26.6)	364 (19.9)	129 (18.8)	577 (24.1)	237 (19.5)	90 (16.2)
35–44	269 (15.4)	408 (22.3)	167 (24.4)	458 (19.1)	218 (18.0)	116 (20.8)
45–54	207 (11.9)	365 (19.9)	149 (21.7)	348 (14.5)	221 (18.1)	135 (24.2)
55–64	159 (9.1)	297 (16.2)	123 (18.0)	223 (9.3)	213 (17.5)	91 (16.4)
65+	207 (11.9)	262 (14.3)	92 (13.4)	348 (14.5)	249 (20.5)	80 (14.3)
Education level						
Primary	254 (14.6)	393 (21.4)	169 (24.7)	346 (14.4)	290 (23.8)	134 (24.1)
Secondary	847 (48.5)	769 (42.0)	328 (48.0)	1018 (42.5)	545 (44.8)	248 (44.5)
Third level	644 (36.9)	669 (36.5)	187 (27.3)	1034 (43.1)	382 (31.4)	175 (31.4)
Social class*						
Social class 1	556 (31.9)	723 (39.5)	206 (30.1)	835 (34.8)	357 (29.3)	153 (27.4)
Social class 2	218 (12.5)	248 (13.5)	95 (13.8)	387 (16.1)	203 (16.7)	94 (16.9)
Social class 3	283 (16.2)	340 (18.6)	130 (18.9)	290 (12.1)	159 (13.1)	61 (11.0)
Social class 4	595 (34.1)	478 (26.1)	221 (32.3)	647 (27.0)	352 (28.9)	185 (33.2)
Social class 5	92 (5.3)	42 (2.3)	33 (4.8)	238 (9.9)	146 (12.0)	64 (11.5)
Employment						
Employed	1,201 (68.8)	1,351 (73.8)	496 (72.5)	1,250 (52.1)	594 (48.8)	256 (46.0)
Unemployed	544 (31.2)	480 (26.2)	188 (27.5)	1,147 (47.9)	623 (51.2)	301 (54.0)
Smoking status						
Current	632 (36.2)	443 (24.2)	180 (26.3)	634 (26.4)	306 (25.2)	138 (24.8)
Former/never	1,113 (63.8)	1,388 (75.8)	504 (73.7)	1,764 (73.6)	911 (74.8)	419 (75.2)
Alcohol intake						
Never	246 (14.1)	256 (14.0)	123 (18.0)	494 (20.6)	316 (26.0)	138 (24.8)
Moderate	685 (39.2)	703 (38.4)	277 (40.4)	1,119 (46.7)	544 (44.7)	300 (53.9)
Frequent	815 (46.7)	872 (47.6)	285 (41.6)	784 (32.7)	357 (29.4)	119 (21.3)

*Note:* All numbers are weighted to account for sampling design; Chronic disease includes lower back pain, osteoarthritis, diabetes, CVD, asthma, bronchitis, anxiety and depression; *Social class (SC) 1 is highest class, SC 4 is lowest, and SC 5 is never worked/unknown/unclassified; P-values (chi-squared): P < 0.0001 for all variables except for social class in females (P = 0.02), employment (P = 0.03 for both genders), smoking in females (P = 0.67), and alcohol in males (P = 0.17).

Univariate analyses were conducted using cross-tabulation to assess the relationship between the prevalence of the various chronic diseases by BMI category. Percentages were compared using a Chi-square test. To examine the association between BMI and the selected chronic diseases, relative risks (RR) and 95% confidence intervals (CI) were estimated using three log-linear binary regression models [16] (defined as Model 1, Model 2, Model 3), using normal weight (BMI 18.5-24.99 kg/m^2^) as the reference category. Model 1 was age-adjusted; Model 2 was adjusted for age, education, social class, and employment; and Model 3 was adjusted for age, education, social class, employment, alcohol and smoking.

To calculate the proportion of risk attributed to overweight and obesity, PAFs for the chronic diseases associated with overweight and obesity were calculated [17,18] using the ‘punaf’ command in Stata. PAFs are estimated from this command using the method recommended by Greenland and Drescher [19]. Punaf calculates CIs for the PAF, and also for scenario means and their ratio. For our ‘ideal’ scenario, overweight and obesity was set to zero. This identifies the proportion of disease that could potentially be prevented if overweight and obesity was eliminated from the population.

For each chronic condition, the relative risk associated with a one kg/m^2^ reduction in BMI was estimated using a log-linear binomial regression that included BMI as a continuous risk factor and adjusted for age, education, social class, employment, alcohol and smoking. These relative risks were combined with the prevalence of each chronic condition to estimate the expected prevalence if each individual’s BMI was reduced by one unit. The change in the prevalence indicates the population level benefit that would be seen for a particular chronic disease if BMI was reduced by one unit for the entire population.

When convergence was not achieved using the log-linear binary model we used a log-linear Poisson model with ‘robust’ estimation variance [20]. Data from participants with a reported BMI of <10 kg/m^2^ (n = 14) or >50 kg/m^2^ (n = 31) were excluded from the analysis in order to remove outliers.

## Results

### Socio-demographic and lifestyle factors

Approximately 49% (n = 4,142) of individuals were in the normal weight category, 36% (n = 3,048) were in the overweight category, and 15% (n = 1,241) were in the obese category (see Table 1). Men were more likely to be overweight (43.0% vs 29.2%) or obese (16.1% vs 13.4%) compared to women. The number of men with two or more chronic conditions increased from 6.9% in the normal weight category to 8.2% in the overweight category and 11.2% in the obese category. The number of women with two or more chronic conditions increased from 9.1% in the normal weight category to 13.0% in the overweight category and 17.4% in the obese category. Overweight and obesity was significantly associated with age (P < 0.0001 for both genders). Normal weight people tended to be younger than those that were overweight or obese. Almost 52% of men and almost 43% of women aged 18–34 years were in the normal weight category. Overweight and obesity was highest in 35–44 year old men. In women, overweight was highest in those aged 65 years and older while obesity was highest in 45–54 year olds. Normal weight people tended to be more educated (P < 0.0001 for both genders). Statistically significant associations were seen between overweight and obesity and social class in men (P < 0.0001) and women (P = 0.02). While overweight people were more likely to be in the highest social class group, obese people were more likely to be in the lowest social class. While overweight and obesity was associated with higher levels of employment in men (P = 0.03), it was associated with higher levels of unemployment in women (P = 0.03). Overweight and obese men were less likely to be current smokers (P < 0.0001), whereas this association was not seen in women (P = 0.67). While normal weight women were more likely to be frequent drinkers (P < 0.0001), this association was not found in men (P = 0.17).

### Gender-specific prevalence of chronic conditions by BMI category

The most prevalent chronic conditions in both men and women were lower back pain, hypertension, and raised cholesterol (Table 2). There was a general trend of increasing prevalence of chronic disease associated with increasing BMI. Increasing BMI was associated with statistically significant increases in the prevalence of lower back pain, osteoarthritis, diabetes and bronchitis in both genders. There were also highly significant increases in the prevalence of hypertension and raised cholesterol associated with increasing BMI in both genders. Asthma, anxiety and depression showed a general trend of increasing prevalence associated with increasing BMI but these were not statistically significant.

**Table 2:** Prevalence of chronic conditions, n (%), according to BMI category and gender

	**Males (n = 4,260)‡**					**Females (n = 4,171)‡**				
**Chronic condition**	**Total prevalence (%)**	**Normal weight**	**Overweight**	**Obese**	P-value*	**Total prevalence (%)**	**Normal weight**	**Overweight**	**Obese**	P-value*
**Lower back pain**	16.2	240 (13.8)	313 (17.2)	135 (19.8)	0.010	16.8	341 (14.3)	220 (18.2)	135 (24.3)	<0.0001
**Osteoarthritis**	3.2	34 (1.9)	67 (3.7)	33 (4.8)	<0.001	6.2	101 (4.2)	102 (8.5)	54 (9.7)	<0.0001
**Diabetes**	3.1	37 (2.1)	59 (3.3)	33 (4.9)	0.0104	2.8	35 (1.5)	43 (3.6)	38 (6.9)	<0.0001
**CVD**	3.7	63 (3.6)	67 (3.6)	25 (3.6)	0.998	2.4	43 (1.8)	46 (3.7)	11 (2.0)	0.010
**Hypertension**	16.3	121 (9.6)	260 (18.3)	144 (26.4)	<0.0001	16.2	161 (9.1)	217 (22.3)	141 (32.1)	<0.0001
**Raised cholesterol**	15.5	114 (9.5)	234 (17.8)	123 (23.8)	<0.0001	18.3	254 (15.6)	184 (20.2)	102 (24.8)	<0.0001
**Asthma**	5.3	79 (4.6)	103 (5.7)	42 (6.2)	0.290	7.2	173 (7.3)	77 (6.4)	49 (8.9)	0.291
**Bronchitis**	2.1	24 (1.4)	44 (2.4)	24 (3.5)	0.011	3.1	67 (2.8)	30 (2.5)	33 (5.9)	0.002
**Anxiety**	4.7	73 (4.2)	91 (5.0)	35 (5.2)	0.553	6.8	148 (6.2)	87 (7.2)	45 (8.1)	0.245
**Depression**	3.9	69 (4.0)	62 (3.4)	33 (4.9)	0.366	6.4	137 (5.7)	79 (6.6)	48 (8.6)	0.060

*Note:***‡** This figure indicates the number of total respondents. As the chronic disease state is not known for all respondents, the actual numbers for each chronic disease does not sum to this figure. Numbers for men ranged from 4,217 to 4,260 with the exception of hypertension (n = 3,225) and raised cholesterol (n = 3,030), and for women the numbers ranged from 4,135 to 4,171 with the exception of hypertension (n = 3,195) and raised cholesterol (n = 2,952). *indicates chi-square p- values.

### Gender-specific associations between chronic conditions and BMI category

Table 3 displays the results of the binary regression model assessing the associations of overweight (BMI 25–29.9 kg/m^2^) and obesity (BMI ≥30 kg/m^2^) with the selected ten chronic conditions among men and women. As the findings for Models 1, 2 and 3 were very similar; the results for Model 3 are presented. Statistically significant associations were found with increasing BMI and a number of chronic conditions, after adjusting for potential confounding factors. A dose–response relationship was seen for a number of conditions with the strongest associations found in obese individuals.

**Table 3:** **Multivariable RRs (95**% **CIs) for selected chronic conditions according to BMI category and gender**

**Chronic condition**	**Males (n = 4,260)‡**		**Females (n = 4,171)‡**	
	**Overweight**	**Obese**	**Overweight**	**Obese**
Lower back pain	1.1 (0.9, 1.3)	1.2 (0.9, 1.5)	1.2 (1.0, 1.4)	1.5 (1.3, 1.9)**
Osteoarthritis	1.5 (1.0, 2.2)	2.0 (1.2, 3.2)*	1.4 (1.0, 1.9)*	1.8 (1.2, 2.5)*
Diabetes	1.1 (0.7, 1.9)	1.6 (0.9, 2.8)	1.9 (1.1, 3.2)*	3.9 (2.5, 6.3)**
CVD	0.8 (0.5, 1.2)	0.7 (0.4, 1.2)	1.7 (1.0, 2.7)*	1.0 (0.5, 2.2)
Hypertension	1.5 (1.2, 1.9)*	2.1 (1.6, 2.7)**	1.9 (1.6, 2.3)**	2.9 (2.3, 3.6)**
Raised cholesterol	1.3 (1.0, 1.7)*	1.7 (1.3, 2.3)**	1.1 (0.9, 1.3)	1.3 (1.0, 1.6)*
Asthma	1.3 (1.0, 1.8)	1.5 (1.0, 2.3)	0.9 (0.6, 1.2)	1.3 (0.9, 1.8)
Bronchitis	1.5 (0.8, 2.5)	1.9 (1.0, 3.5)	0.7 (0.5, 1.1)	1.7 (1.0, 2.9)*
Anxiety	1.1 (0.8, 1.7)	1.1 (0.7, 1.7)	1.0 (0.8, 1.4)	1.1 (0.8, 1.5)
Depression	0.8 (0.5, 1.2)	1.0 (0.6, 1.5)	1.1 (0.8, 1.4)	1.3 (0.9, 1.8)

*Note:* Figures adjusted for age, education level, social class, employment status, alcohol intake and smoking status; **‡** This figure indicates the number of total respondents. As the chronic disease state is not known for all respondents, the actual numbers for each chronic disease does not sum to this figure (see similar note in Table 2); *P-value <0.05; **P-value <0.001.

There was a positive association between increased BMI and lower back pain although this association was statistically significant in obese women only (RR 1.5; 95% CI 1.3-1.9). For osteoarthritis, obese men had twice the risk compared to normal weight men (95% CI 1.2-3.2). In women, there were positive associations with osteoarthritis for both overweight (RR 1.4; 95% CI 1.0-1.9) and obesity (RR 1.8; 95% CI 1.2-2.5). For diabetes, although BMI showed a graded increase, this increase was not statistically significant in either overweight or obese men compared to men in the normal weight category. However, there was a strong statistically significant association between diabetes and increasing BMI in women. The risk of diabetes in overweight women was almost twice that of normal weight women (95% CI 1.1-3.2), and the risk in the obese category increased almost four- fold compared to women in the normal weight category (95% CI 2.5-6.3). For CVD, a positive statistically significant association was found in overweight women only (RR 1.7; 95% CI 1.0-2.7). Results for overweight and obese men were suggestive of a protective effect against CVD but these were not statistically significant. Hypertension and raised cholesterol showed graded and mostly significant associations with increasing BMI for both genders. These associations were stronger for hypertension than for raised cholesterol. The association with hypertension was stronger in women compared to men both in the overweight (RR 1.9 vs. RR 1.5) and obese (RR 2.9 vs. RR 2.1) groups. Conversely, the association with raised cholesterol was stronger in men compared to women both in the overweight (RR 1.3 vs. 1.1) and obese (RR 1.7 vs. 1.3) groups. Asthma showed no statistically significant associations with increased BMI for both genders, although the relationship was graded in men. There was an association between increased BMI and chronic bronchitis although this was statistically significant in obese women only (RR 1.7; 95% CI 1.0-2.9). In both genders, there were no statistically significant associations found between anxiety and depression and increasing BMI.

### Gender-specific PAFs for selected chronic conditions attributable to overweight and obesity

**Figure 1:**
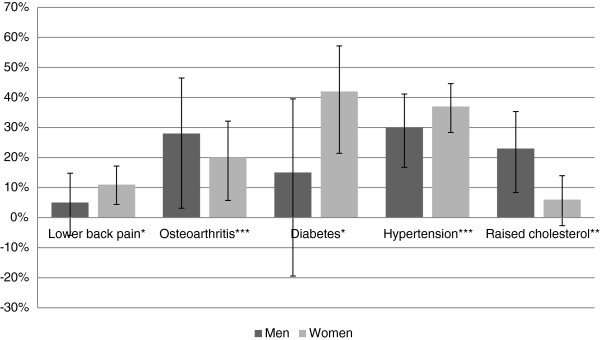
**Population attributable fractions (%) of selected chronic conditions attributable to overweight and obesity (BMI ≥25 kg/m**^**2**^**) by gender.** Note: The vertical lines represent 95% confidence intervals; *p value < 0.05 for women only; **p value < 0.05 for men only; ***p value < 0.05 for men and women.

Overweight and obesity contribute significantly to the burden of a number of chronic diseases (Figure 1). Overall, the burden of disease associated with overweight and obesity was higher among women. In women, the largest burden of disease associated with overweight and obesity, and the proportion of disease that could potentially be prevented if overweight and obesity was eliminated from the population includes 42% (95% CI 22%-58%) of diabetes, 37% (95% CI 29%-45%) of hypertension, 20% (95% CI 6%-32%) of osteoarthritis, and 11% (95% CI 5%-18%) of lower back pain. In men, the proportion of disease that could potentially be prevented if overweight and obesity was eliminated from the population includes 30% (95% CI 17%-41%) of hypertension, 28% (95% CI 3%-46%) of osteoarthritis, and 23% (95% CI 8%-35%) of raised cholesterol. Although increases were seen for diabetes and lower back pain in men, these were not statistically significant.

### Reduction in prevalence of selected chronic conditions associated with a one-unit reduction in BMI

Table 4 presents the expected risk reduction for the selected chronic conditions associated with a one unit (1 kg/m^2^) decrease in BMI, stratified by gender and adjusted for the variables as in Table 3. Overall, by lowering BMI by one unit across the population, in analyses of both genders combined, it is expected that there would be 28 fewer cases of chronic disease (here chronic disease includes hypertension, raised cholesterol, lower back pain, osteoarthritis, diabetes, and asthma) per 1,000 population. Broken down by gender, it is expected that overall there would be a reduction in chronic disease of 26 (4%) and 28 (4%) cases per 1,000 population for men and women respectively. The greatest reduction in cases of chronic disease associated with a population-wide one unit decrease in BMI was found for hypertension with a reduction of 11 (7%) and 12 (7%) cases per 1,000 men and women respectively. This is followed by raised cholesterol, with a greater reduction expected in men. Compared to men, there is a greater reduction in cases of lower back pain, osteoarthritis and diabetes expected in women, while there is a greater reduction in cases of asthma expected in men.

**Table 4:** **Reduction (n,%) per 1,000 population in selected chronic conditions associated with a 1 kg/m**^**2 **^**reduction in BMI**

	**Males**			**Females**		
**Chronic condition**	**Scenario 1**^**a **^**(n, per 1,000)**	**Scenario 2**^**b **^**(n, per 1,000)**	**Reduction**^**c **^**n (%)**	**Scenario 1**^**a **^**(n, per 1,000)**	**Scenario 2**^**b **^**(n, per 1,000)**	**Reduction**^**c **^**n (%)**
**Hypertension**	162.6	151.9	10.7 (6.6)	162.4	150.7	11.7 (7.2)
**Raised cholesterol**	154.9	146.7	8.2 (5.3)	183.3	179.5	3.8 (2.1)
**Lower back pain**	162.1	159.3	2.8 (1.7)	168.2	162.6	5.6 (3.3)
**Osteoarthritis**	31.5	30.1	1.4 (4.0)	62	58.9	3.1 (5.0)
**Diabetes**	30.6	29.5	1.1 (3.6)	28	25.5	2.5 (8.9)
**Asthma**	52.9	50.9	2.0 (3.8)	72.1	70.6	1.5 (2.1)

*Note:*^a^Prevalence of chronic disease based on current BMI levels; ^b^Predicted prevalence of chronic disease based on a one unit (1 kg/m2) reduction in population BMI, estimated by dividing the prevalence estimates (Scenario 1) by the relative risks obtained from a log-linear regression using BMI as a continuous variable; ^c^The reduction in chronic disease prevalence in the population associated with a one unit reduction in BMI.

Extrapolated to the 2011 Republic of Ireland population (CSO 2011 population: Male 18+ years = 1,684,456; Female 18+ years = 1,745,919) [21], following a one unit population-wide reduction in BMI, it is expected that overall there would be 44,133 fewer cases of chronic disease in men and 49,235 fewer cases of chronic disease in women. The greatest reduction in cases of disease associated with a population-wide one unit decrease in BMI is expected for hypertension with 18,024 fewer cases in men and 20,427 fewer cases in women.

We also investigated the expected prevalence of overweight and obesity associated with a one unit reduction in BMI. If the population BMI reduced by one unit, the current prevalence estimates of overweight would reduce from 43.0% to 37.2% in men and from 29.2% to 23.9% in women. The current prevalence estimates of obesity would reduce from 16.1% to 11.6% in men and from 13.4% to 10.2% in women.

## Discussion

This study examined the association between overweight and obesity and several chronic diseases using nationally representative survey data from the Republic of Ireland. It can be concluded from the results that firstly, there is a high prevalence of overweight and obesity (BMI ≥25 kg/m^2^) in Irish adults, particularly among men (59% vs. 42%). Secondly, overweight and obesity is a major contributor to a range of chronic diseases and carries a significant disease burden in the Republic of Ireland, particularly among women. A small reduction in BMI at a population level would potentially lead to substantial gains in terms of reduced prevalence of chronic disease.

In this study, hypertension and raised cholesterol in men, and osteoarthritis, diabetes, CVD, and hypertension in women were significantly more prevalent in the overweight category. In the obese category, statistically significant associations were observed for osteoarthritis, hypertension, and raised cholesterol in men, and for lower back pain, osteoarthritis, diabetes, hypertension, raised cholesterol, and chronic bronchitis in women. As the RRs generally increased with increasing BMI, this implies a direct association between increasing BMI and increasing prevalence of related chronic disease. Previous cross-sectional studies assessing the overall burden of chronic disease show similar findings for a number of conditions [22-25]. Although most of the following did not reach statistical significance, graded associations were generally seen for lower back pain, asthma, chronic bronchitis, anxiety, and depression. Lack of a statistically significant association between overweight and obesity and diabetes in men may reflect weight loss following diagnosis. Similar considerations may apply in relation to the apparently negative association with CVD, along with the small number of study participants with this condition.

The PAFs indicated that a large proportion of a number of chronic diseases are attributed to overweight and obesity, suggesting that obesity is an important cause of morbidity, with a significant impact on health care costs. A recent report [26] highlighted the substantial direct and indirect costs associated with overweight and obesity in the Republic of Ireland, with 2009 estimates at €1.13 billion. Direct healthcare costs accounted for 35% and indirect costs accounted for 65% of the total costs. As there is a high level of indirect costs associated with chronic diseases such as lower back pain and osteoarthritis, and direct costs associated with chronic diseases such as CVD, reductions in these diseases are likely to reduce costs incurred with obesity.

As the importance of population-based strategies has long been recognised [27], a one unit population reduction in BMI was assessed to estimate the effect such a strategy may have on the prevalence of chronic disease. As the prevalence of overweight and obesity is high, and a considerable proportion of the population are at risk of a number of chronic diseases, the population approach targeting the entire population is likely to be more effective and potentially less costly than targeting high-risk individuals, in reducing the prevalence of overweight and obesity in the population and thus the burden of disease attributable to overweight and obesity. In men, there was a 4% reduction in chronic disease (reduced from 595 to 568 cases per 1,000 men) associated with a one unit population-wide decrease in BMI. In women, there was a 4% reduction in chronic disease (reduced from 676 to 648 cases per 1,000 women) associated with a one unit population-wide decrease in BMI.

A number of limitations need to be taken into account. The cross-sectional study design cannot provide evidence of a temporal relationship or causality. Findings on associations with individual conditions such as CVD and diabetes must be interpreted cautiously given the potential for reverse causation. Compared to longitudinal studies, risk estimates are likely to be reduced [24]. Thus, estimates in this study may under-represent the actual risk of developing a disease. Although the SLÁN 2007 survey had a reasonable response rate (62%), there is the potential for selection bias, for example, less healthy individuals may be more likely to refuse to participate [28], which may have resulted in an underestimated prevalence of overweight and obesity and of chronic disease.

The use of self-reported height and weight may have resulted in reporting bias. Evidence suggests that a considerable number of Irish adults underestimate their body weight [13], therefore, prevalence estimates in this study are likely to be underestimated. There may also have been sex differences in the reporting of height and weight. While there is a general trend of under-reporting for weight and over-reporting for height, the degree of this trend varies for men and women [29]. One study found that while females tended to underestimate their weight, males were inclined to slightly overestimate their weight [30]. A recent study examining trends in misclassification patterns of measured and self-reported BMI in the SLÁN surveys did not find a trend related to gender bias [31], suggesting that the extent of sex differences in the reporting of BMI in this study is not likely to be considerable. The use of self-reported chronic conditions may also have resulted in reporting bias, resulting in an underestimation of the true prevalence of chronic disease and thus an underestimation of the strength of their association with overweight and obesity. Similar to the reporting of height and weight, there may also have been sex differences in the reporting of chronic conditions; women have been shown to more accurately self-report diagnoses compared to men [32]. Nonetheless, it has been found that there is considerable agreement between medical record diagnosis of disease and patients' self-reports for a number of chronic diseases [32,33]. The known association between excess weight and chronic conditions increases the likelihood of diagnosis in heavier people and may represent an additional source of bias. For example, it is likely that diagnosis of high blood pressure and raised cholesterol is greater in overweight and obese individuals due to more frequent measurements in these individuals compared to those that are of normal weight.

The use of BMI as a measure of excess body weight may lead to some misclassification as it does not distinguish between fat and muscle mass [13]. The use of other methods including waist circumference, waist-to-hip ratio, and skin-fold thickness may provide more accurate estimates, however, these were not available in the SLÁN dataset.

It must be noted that for many specific conditions, such as CVD, the survey produced a relatively small number of cases, thus possible associations may have been missed. The findings on individual conditions are constrained by limited statistical power and random sampling error. Furthermore, the burden of conditions with a short natural history for which there are known associations with overweight and obesity, such as a number of major cancers, cannot be captured in this type of study. Therefore, the overall burden of disease associated with overweight and obesity is underestimated.

Despite these limitations, the current analyses highlight and quantify the burden of prevalent chronic disease associated with overweight and obesity and the findings are similar to other nationally representative cross-sectional studies [34-36]. The results of this study are applicable at a population level as a result of applying sampling weights. A number of important potential confounding factors were adjusted for in the analyses and a number of models were employed to assess potential differences in association depending on the adjustment of certain factors. PAFs, which are useful for informing public health interventions, were calculated, although some authors argue that in order to inform public health interventions, such interventions should be precisely defined in the estimations [37]. A one unit reduction in BMI was assessed to strengthen the evidence of the burden of chronic disease associated with overweight and obesity and the potential decrease in this burden if overweight and obesity was reduced or eliminated in the population.

## Conclusions

The findings of this study support previous observations of a positive association between overweight and obesity and a number of chronic diseases. The results highlight that overweight and obesity are major contributors to the burden of chronic disease in the Irish population. Further research in this area will benefit from improved datasets, data collections methods and study designs. These efforts will help achieve more reliable comprehensive estimates than could be achieved within the scope of this study. Due to the scale of the problem of overweight and obesity, population-based strategies for the prevention of overweight and obesity are urgently needed. Achieving a relatively modest reduction in average BMI at the population level is likely to significantly impact the burden of disease attributable to overweight and obesity. Further research, focused public health strategies, and political commitment will likely reduce the prevalence of overweight and obesity and the burden of associated disease, resulting in improved population health and reduced burden on the health service in the future.

## Abbreviations

BMI: Body mass index; CVD: Cardiovascular disease; PAF: Population attributable fraction; RR: Relative risk; SLÁN: Survey of lifestyle, attitudes and nutrition.

## Competing interests

The authors declare that they have no competing interests.

## Authors’ contributions

AD and IJP proposed the study concept. KK participated in the design of the study and drafted the original manuscript. ED assisted with statistical analyses. KK and APF conducted the statistical analyses. All authors contributed to the drafting and editing of the manuscript, provided feedback and critique of the study design and approach, and approved the final manuscript.

## Pre-publication history

The pre-publication history for this paper can be accessed here:

http://www.biomedcentral.com/1471-2458/14/143/prepub
